# Egg capsule mineralization via vaterite transportation in the invasive apple snail *Pomacea canaliculata*

**DOI:** 10.1093/molbev/msag164

**Published:** 2026-07-07

**Authors:** Jingliang Huang, Li Li, Haohua Zhang, Ruoxi Du

**Affiliations:** Southern Marine Science and Engineering Guangdong Laboratory (Guangzhou), Guangzhou 511458, China; School of Chemical Engineering and Technology, Sun Yat-sen University, Zhuhai 519082, China; School of Life Science, Tsinghua University, Beijing 100084, China; Southern Marine Science and Engineering Guangdong Laboratory (Guangzhou), Guangzhou 511458, China; Ocean University of China, Sanya Oceanog Inst, Sanya 572025, China

**Keywords:** *Pomacea canaliculata*, egg capsule, vaterite, organic matrix, proteomics

## Abstract

The invasive apple snail *Pomacea canaliculata* utilizes calcified egg capsules as a key adaptation for terrestrial reproduction; however, the biomineralization mechanisms underlying capsule formation remain poorly understood. In this study, we found that vaterite, a rare calcium carbonate polymorph, was deposited in the egg capsule through a unique transport and assembly process. We demonstrated that calcium carbonate nanoparticles (several hundreds of nanometers in diameter) were initially stored in the egg yolk and subsequently transported to the capsule surface, where they formed a protective vaterite layer (around 10 μm). Proteomic and transcriptomic analyses identified a specialized organic matrix. This matrix contains chitin-binding proteins (CBPs), sulfatases, and calcium-binding proteins, which act collectively stabilize vaterite and inhibit calcite formation. Phylogenetic analysis suggested CBPs represent a group of evolutionarily conserved yet functionally versatile secretory proteins, distinct from shell-specific proteins like Pif, highlighting the snail’s ability to repurpose existing genes for novel mineralization. Furthermore, gland-specific transcriptomics revealed upregulated pathways in mineral absorption and glycosaminoglycan biosynthesis, underscoring the coordinated roles of the albumen and capsule glands in matrix production. These findings not only elucidate a unique biomineralization strategy in the apple snail but also identify potential molecular targets for disrupting capsule formation, offering new avenues for controlling this globally invasive species.

## Introduction

The apple snail *Pomacea canaliculata*, native to South America, has emerged as a highly invasive species across multiple continents due to its rapid reproductive capacity and lack of natural predators in new environments (Hayes et al. 2015; Panda et al. 2021). As an amphibious species, *P. canaliculata* thrives in freshwater habitats where they feed voraciously on aquatic crops including rice, *Zizania latifolia*, and *Trapa bispinosa*, causing significant agricultural damage (Azmi et al. 2022; Yin et al. 2022). Beyond its economic impact, this species serves as an intermediate host for dangerous parasites such as *Angiostrongylus cantonensis*, posing serious public health risks (Lv et al. 2020). The species also causes ecological disturbances through acidic excretions and predation on juvenile native snails (Jiang et al. 2022). These multifaceted threats underscore the urgent need for understanding the mechanisms behind the snail's reproductive adaptation to develop effective control strategies that target its unique biological characteristics.

A key factor in *P. canaliculata*'s invasion success lies in its remarkable reproductive strategy involving terrestrial egg deposition. As an amphibious species, adults of *P. canaliculata* inhabit freshwater environments while embryos develop terrestrially, with females laying their eggs above water on vegetation or other substrates. However, terrestrially developed embryos face distinct environmental challenges, including desiccation, temperature fluctuations, and predation (Turner 1998). The snail has evolved 2 crucial adaptations to overcome these challenges: a nutrient-rich perivitelline fluid that provides hydration and antioxidant protection, and a calcified egg capsule that serves multiple protective functions. This specialized capsule, composed primarily of vaterite, forms a physical barrier against pathogens and UV radiation, while maintaining gas exchange through its intricate micro-nano channel structure (Dreon et al. 2006). The capsule's calcium carbonate content not only provides structural integrity but also serves as a mineral reservoir for developing embryos (Heras et al. 1998). Moreover, with an extended breeding season allowing multiple oviposition events, this species can annually produce thousands of eggs with a hatching rate over 90% (Hayes et al. 2015). Such a highly efficient reproductive strategy has been found in another invasive gastropod, the giant African snail *Achatina fulica*, which produces eggs encapsulated by a calcitic layer (Patiño-Montoya et al. 2024). Biomineralization also plays an important role in the adaptation of many other invasive species to local habitats, such as the golden mussel *Limnoperna fortune* with calcified shells, sponges with calcium carbonate spines for adhesion and growth (Longo et al. 2012; Boltovskoy et al. 2025). Therefore, elucidating the biomineralization process in invasive species could aid in developing control strategies to limit their spread.

Previous studies in the biomineralization field mainly focus on the molluscan external shells and avian eggshells, which contain thermostable calcium carbonates. In molluscs, the mineralized shells, usually composed of calcitic outer layers and aragonitic inner layers, are tightly regulated by the embedded polysaccharides and proteins, with the latter playing vital roles in directing the nucleation, growth, and polymorph selection of calcium carbonate crystals (Fang et al. 2012; Pan et al. 2014). Identifications of the shell proteins reveal that proteins having the von Willebrand A (vWA) domain and chitin-binding affinity are prevalent in various genera (Carini et al. 2019; Liu et al. 2023a). Similarly, uterine proteins and mucins in avian eggshells are found to organize the calcitic microstructure that ensures the mechanical protection and gas exchange during embryo development (Sun et al. 2013; Le Roy et al. 2021). Vaterite biominerals are less common in nature due to their thermal instability, and are only found in a few instances, such as in abnormal deposits in freshwater pearls and in the sclerites of the soft coral *Ovabunda macrospiculata* (Xeniidae) (Qiao et al. 2008; Drake et al. 2021). The exclusive use of vaterite for constructing egg capsules in apple snails is unique, yet the molecular mechanism behind this phenomenon remains unclear, though it has been described for many years (Hall and Taylor 1971).

The egg capsule mineralizes via a 2-stage process (in vivo and ex vivo), with the in vivo stage being well characterized. During the initial internal stage, fertilized eggs become enveloped in perivitelline fluid and egg jelly within the specialized albumen gland-capsule gland complex (Mu et al. 2017). The albumen gland plays a dual role, containing abundant vaterite particles that serve as the primary calcium source (Meenakshi et al. 1974) and featuring 2 distinct cell types: albumen secretory cells that produce perivitelline fluid proteins, and labyrinthine cells specialized for calcium deposition and transport (Catalán et al. 2002). Simultaneously, the capsule gland secretes a complex organic matrix containing mineralization-related proteins and polysaccharides that guide the calcification process. Transmission electron microscope (TEM) and histochemical analysis demonstrated that these secretory cells exhibit remarkable functional specialization, contributing to the egg jelly's multilayered structure and ultimately determining the capsule's microstructure (Catalán et al. 2002).

Following oviposition, the external stage begins, during which calcium carbonates are deposited on the egg surface. Although *P. canaliculata* has been extensively studied for its reproductive biology (Cadierno et al. 2018; Azmi et al. 2022; Yin et al. 2022) and its genome had been sequenced in 2018 (Liu et al. 2018), significant gaps remain in understanding its unique biomineralization process, particularly the ex vivo calcification stage. In this study, we examined the egg capsule calcification by focusing on the early stage of the oviposition and the source of vaterite. These investigations not only advance fundamental knowledge of molluscan biomineralization but may also reveal novel targets for species-specific control.

## Materials and methods

### Sample collection

Individuals of the apple snail *P. canaliculata* and their egg masses were collected from freshwater ponds in the Zhuhai Campus of Sun Yat-sen University. Specifically, freshly laid egg masses and egg-laying females were collected at night and immediately frozen in liquid nitrogen. The frozen samples were stored at −80 °C until used.

### Scanning electron microscope (SEM) observation

The albumen glands and egg masses were freeze-dried using a freeze-dryer. The dried samples were placed on copper stubs and coated with platinum. A scanning electron microscope equipped with an energy-dispersive X-ray spectroscopy (EDS) detector (JEOL, Japan) was applied to examine the microstructure and elemental composition. The accelerating voltage was 15 kV under high vacuum conditions. Calcium carbonates in the in vitro CaCO_3_ crystallization experiment were measured with the same method.

### Mineralogy determination

The mineralogy of the egg capsule samples was determined by powder X-ray diffraction (Rigaku Ultima IV, Japan). The scanning angle (2*θ*) ranged from 5˚ to 80˚ with a scanning speed of 10˚ per min. For crystals in an *in vitro* crystallization experiment, the mineralogy of the crystals was determined by Raman spectroscopy (Renishaw inVia, UK) with a scanning range of 100 to 1,800 cm^−1^. The 532 nm laser device was applied, and the laser power was set to 10%, and the data were collected for 1 s with 2 scans. Standard X-ray diffraction (XRD) spectra of vaterite were obtained from the American Mineralogist Crystal Structure Database (https://rruff.info/) and calcite at the International Centre for Diffraction Data (https://www.icdd.com/pdfsearch/).

### Organic matrix extraction

Egg masses of *P. canaliculata* were mechanically disrupted to break the capsules and immersed in 5% sodium hypochlorite (NaClO) solution for 12 h to remove residual egg yolk. Samples were subsequently air-dried, ground into fine powders and immersed in 5% NaClO solution for another 12 h to further eliminate protein contamination from cell debris and cytoplasm. This treatment likely removes surface-associated biomolecules, potentially leading to an underestimation of the total capsule matrix content. However, as shown in the following Results section, there should be some internal stabilizer of vaterite embedded in the crystals, and we mainly focus on this part of the organic matrix. The resulting capsule powder was completely decalcified in 0.5 M ethylenediaminetetraacetic acid (EDTA) at 4 °C under continuous stirring for 48 h. The mixture was centrifuged to separate the pellets (designated as EDTA-insoluble capsule matrix, EICM) and the supernatant (designated as EDTA-soluble capsule matrix, ECM). The ECM fraction was dialyzed against ddH_2_O at 4 °C using dialysis bags with a molecular weight cut-off of 3 kDa, with ddH_2_O changed 3 times every 12 h. The purified ECM was concentrated using an ultrafiltration device (molecular weight cut-off of 3 kDa).

### Characterization of egg capsule matrix

The prepared EICM and ECM were analyzed by sodium dodecyl sulfate-polyacrylamide gel electrophoresis using standard procedures. Gels were stained with Coomassie Brilliant Blue, and the gel lanes corresponding to the EICM and ECM were cut and analyzed with LC-MS/MS in Tsinghua University Analysis Centre (Beijing, China) according to the method described in our previous study (Huang et al. 2021). In brief, the gel lanes were destained in a 50 mM NH_4_HCO_3_/acetonitrile (50/50) mixture and immersed in 50 mM NH_4_HCO_3_ solution containing 10 mM DTT for reduction. Subsequent alkylation was accomplished by treating with 100 mM iodoacetamide at room temperature. Then, the gel pieces were dried in acetonitrile and digested with trypsin. The LTQ Orbitrap Velos mass spectrometer with a Dionex U-3000 Rapid Separation nano-LC system (Thermo Scientific) was used to analyze the digest peptides. The Analyst QS 1.1 software (Applied Biosystems) was used to collect MS data, and the MS survey scan was over m/z 350 to 1,500. Raw data were searched against protein databases derived from *P. canaliculata* in NCBI. Proteins with score >2 and unique peptides >2 were considered solid. Signal peptides were predicted via the online search engine SignalP5.0 (https://services.healthtech.dtu.dk/services/SignalP-5.0/). Domain arrangement was analyzed using SMART (https://smart.embl.de/).

Fourier transform infrared spectroscopy (FTIR) of the EICM was performed as previously described (Huang et al. 2021).

### *In vitro* CaCO_3_ crystallization

*In vitro* CaCO_3_ crystallization experiments were performed to explore the effect of capsule matrix on CaCO_3_ precipitation. In brief, the reaction solution was prepared by mixing pre-cooled CaCl_2_ stock and the concentrated capsule ECM, followed by adding NaHCO_3_ solution and mixing. Then, the mixed solution was dropped onto cover glasses and placed in a desiccator. The desiccator was placed in the dark at room temperature for 24 h. After incubation, the cover glasses were gently rinsed with ddH_2_O and air-dried.

### RNA extraction and high-throughput RNA-seq

Tissues (50 to 100 mg) were frozen in liquid nitrogen and homogenized with an electric tissue grinder. Total RNA was extracted using the Total RNA Extraction Reagent (TAKARA, Japan), followed by the removal of genomic DNA with DNase I. The quality and concentration of the purified RNA were assessed using a Qubit Fluorometer (Thermo Fisher Scientific, United States) and a NanoDrop 2000 (Thermo Fisher Scientific, United States), respectively. RNA integrity was evaluated via agarose gel electrophoresis and Q-seq1. PE150 high-throughput sequencing was conducted on the Illumina Novaseq 6000 platform. To compare gene expression levels across samples, RNA-seq data were processed using edgeR. Differentially expressed genes (DEGs) were defined with the criteria of false discovery rate < 0.05 and |fold change| > 2. These DEGs were subsequently annotated through Kyoto Encyclopedia of Genes and Genomes (KEGG) clustering and enrichment analysis.

### Phylogenetic tree construction

Chitin-binding protein (CBP) homologs in other molluscs were searched in NCBI with the BLAST tool (https://blast.ncbi.nlm.nih.gov/Blast.cgi). For the Aplacophora *Chaetoderma* sp., the protein database was obtained from http://mgbase.qnlm.ac/, and TBtools was used to search homologs (Chen et al. 2020). The MEGA (version 12.0) tool was used for the phylogenetic tree construction with the neighbor joining method. Besides the 4 identified CBPs in the apple snail *P. canaliculata*, CBPs from 18 species were selected for analysis, including 15 molluscs (*Liolophura sinensis*, *Crassostrea virginica*, *Octopus sinensis*, *Batillaria attramentaria*, *Littorina saxatilis*, *Dreissena polymorpha*, *Elysia crispata*, *Mya arenaria*, *Ruditapes philippinarum*, *Haliotis rufescens*, *Gigantopelta aegis*, *Mytilus coruscus*, *Chaetoderma* sp., *Pinctada fucata*, *Pictodentalium vernedei*), 1 starfish *Acanthaster planci*, 1 brachiopod *Lingula anatina*, and 1 sponge *Ephydatia muelleri*.

### Data analysis and image processing

Excel version 2021 (Microsoft, Redmond, United States) and TBtools (Chen et al. 2020) were used to perform basic bioinformatic analysis of the omics data. The FTIR and TGA data were visualized by OriginLab version 2021 (OriginLab Corporation, Northampton, United States). Imaged figures were processed using Adobe Photoshop CS6 (Adobe Systems, San Jose, California).

## Results

### Microstructure and composition of the egg capsules

**Figure 1:**
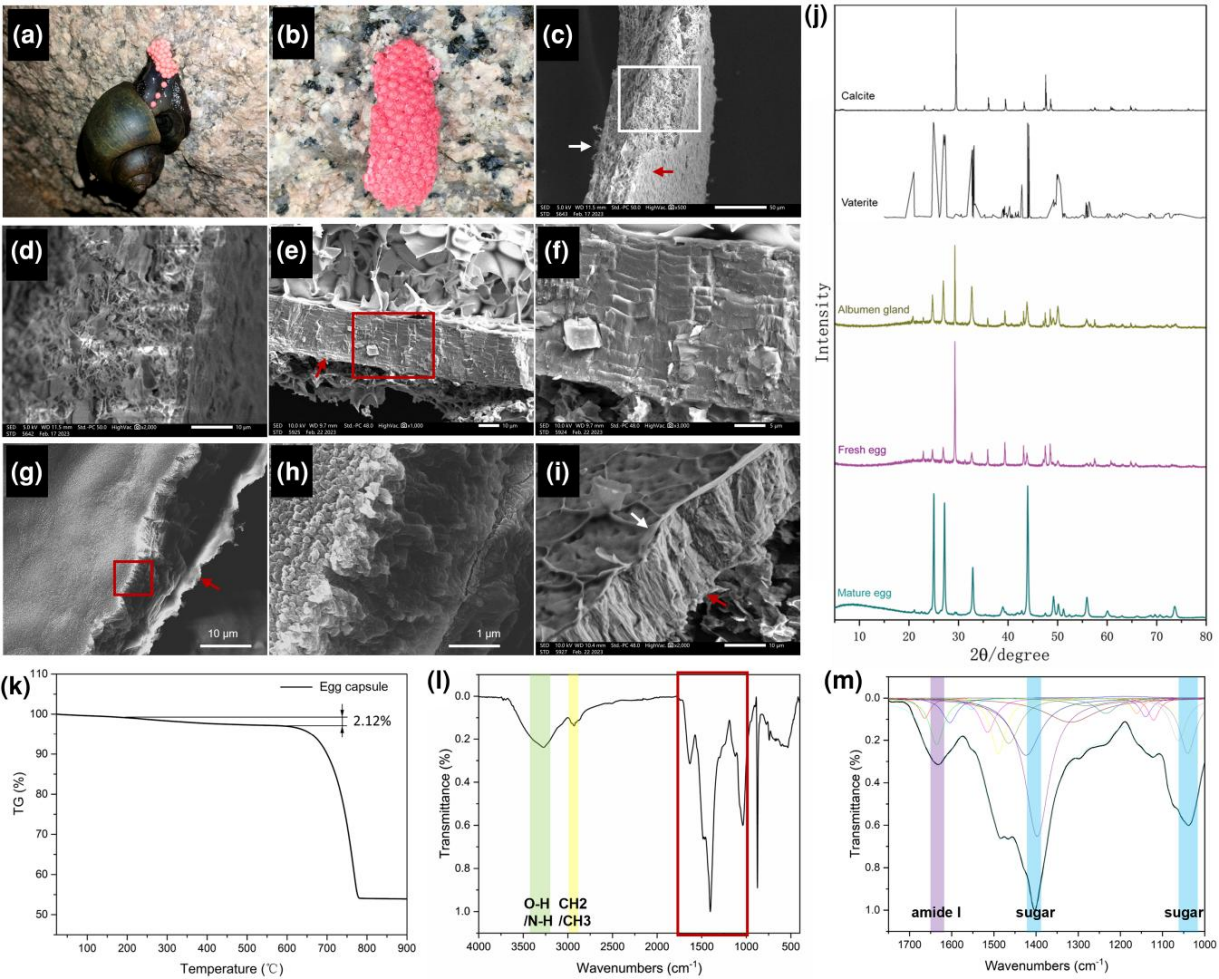
Microstructure and composition of the egg capsule in *P. canaliculata*. a) and b) The egg-laying female and mature egg mass; c) and d) the SEM observation of a newly laid egg; (d) is an enlarged view of (c) (white frame). e) and f) The microstructure of a fully mineralized egg capsule; (f) is an enlarged view of (e) (red frame). g) and h) Mature capsules treated with NaClO solution; (h) is an enlarged view of (g) (red frame). i) An untreated capsule with a similar microstructure to (h) but different to (f). j) XRD analysis of 3 CaCO_3_ samples from albumen gland, fresh eggs, and mature eggs. Calcite (RRUFFID = R040070) and vaterite (amcsd 0019866) standards were obtained from the public database RRUFF. k) TGA analysis of the egg capsule of *P. canaliculata*. The weight loss between 200 and 600 °C is due to the decomposition of organic matter. l) and m) FTIR spectra of the organic matrix in the egg capsule; m is an enlarged view of the red dashed box in (l). In (m) multiple peak fit was performed to figure out the complex feature of the matrix.

The apple snail *P. canaliculata* usually climbs out of water and lays eggs on terrestrial surfaces from sunset to midnight. The freshly laid eggs appeared as soft spheres embedded within a transparent gel which also facilitated the adhesion of the egg mass to the terrestrial substrates such as rocks or plants (Fig. 1a). The surface of each egg gradually mineralizes and forms a calcified capsule which protects the zygote and egg yolk from physical damage and desiccation (Fig. 1b). We verified the dehydration-preventing effect of the capsule by partially decalcifying the capsule with EDTA. The egg masses lacking an intact egg capsule exhibited progressive weight loss (Figure S1a), and the eggs failed to hatch (Figure S1b), indicating that the calcified capsule plays an important role in maintaining the water content inside the eggs.

We applied SEM to examine the microstructure of the egg capsule. It was found that the surface of newly laid eggs was covered by a loosely packed organic matrix approximately 50 μm in thickness (Fig. 1c and d). In mature eggs, a 20 μm-thick mineralized layer was observed, consisting of orderly deposited minerals (Fig. 1e and f). XRD analysis confirmed its composition of pure vaterite (Fig. 1j), consistent with previous studies in other *Pomacea* species (Hall and Taylor 1971; Meenakshi et al. 1974). In Fig. 1e, the capsule exhibited horizontally stacked tablets, similar to the nacre microstructure in Pterioida (Marie et al. 2017; Muhammad et al. 2017). Interestingly, in some specimens, the vaterite were arranged vertically, perpendicular to the egg surface (Fig. 1g to i), and numerous nano channels were observed after removal of organic matter after NaClO treatment (Fig. 1h). Unlike the densely packed molluscan shells (including *P. canaliculata* shell), these nano pores may facilitate gas exchange between the egg and the ambient environment. The complex microstructures of the egg capsule may be related to various functions, indicating that the capsule mineralization process is finely regulated.

**Figure 2:**
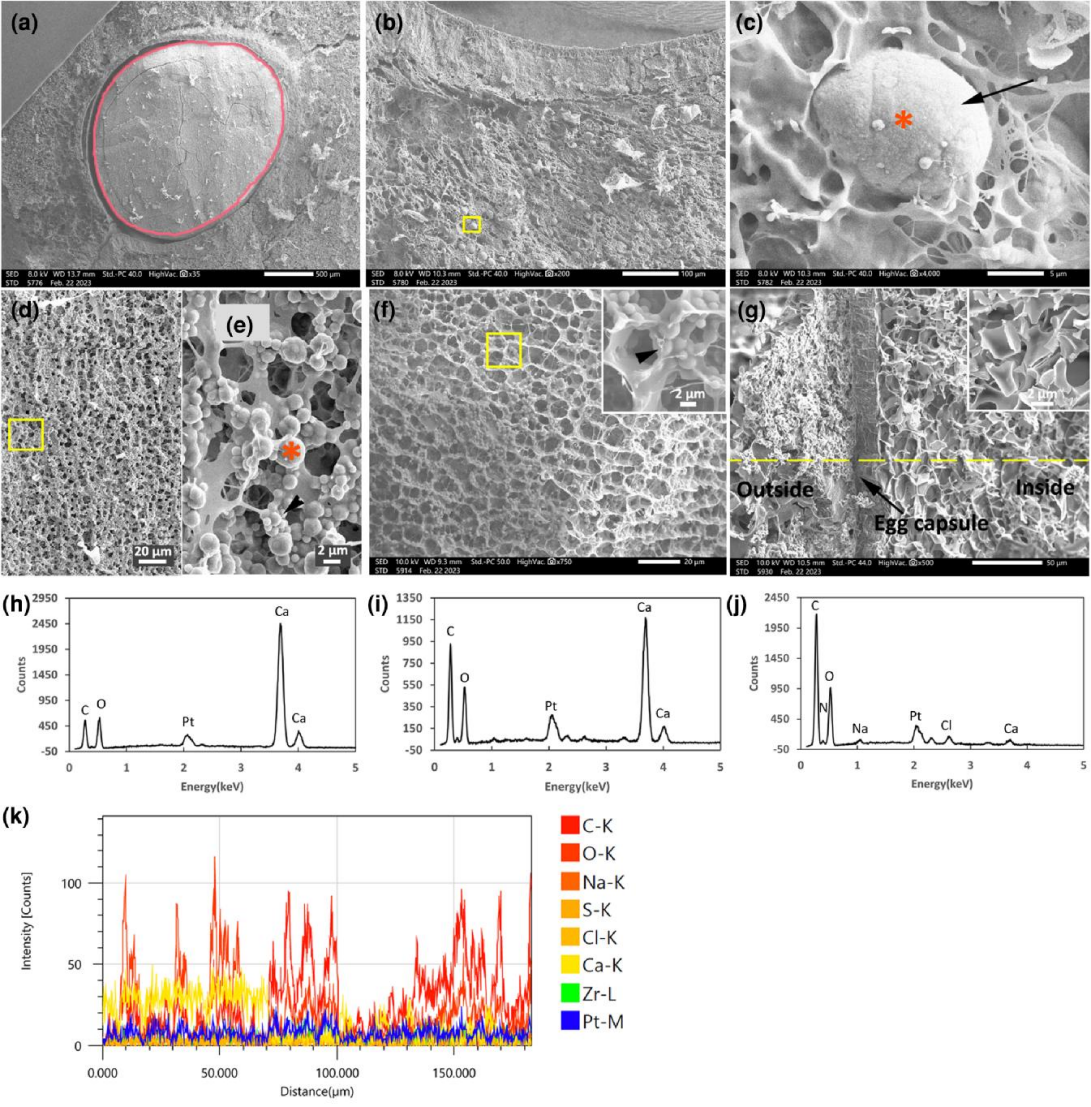
SEM observation revealed the transportation of calcium carbonate during egg capsule formation. a) to g) Cryo-dried samples. a) A secreting egg (pink circle) passing through the capsule gland duct. b) Tissues of the albumen gland and capsule gland surrounding the egg. c) An enlarged view of the yellow box in (b), showing a calcium carbonate particle (black arrow). d) and e) The egg yolk in the capsule gland duct at different magnifications. f) The internal structure of a newly laid egg, with the inset at the upper right corner being an enlarged view of the yellow frame. g) The internal structure of a mature egg, with the inset at the upper left corner being an enlarged view of the yellow frame. h) and i) EDS analyses of the particles (red asterisks) in (c) and (e), respectively. j) The full-area EDS analysis result of the inset in (g). k) The EDS line scan result at the yellow dashed line in (g), with the zero point being the beginning of the yellow dashed line. The Pt signal in the EDS analysis was due to the coating platinum during the sample preparation process.

Previous studies suggest that biominerals are deposited under the control of organic matrices (Marie et al. 2017; Rivera-Pérez and Hernández-Saavedra 2021). TGA results showed that the egg capsule contains more than 97% calcium carbonate and a small amount of organic matrix (around 2.12%) (Fig. 1k). It should be noted that we prepared the capsule sample by removing the egg yolk with sodium hypochlorite because the capsule is too small and fragile, so the total matrix content may be under-estimated. As revealed by the FTIR spectra, the extracted capsule matrix was a mixture of organic materials, so we performed multiple peak fits of the spectrum (1,800 to 1,000 cm^−1^) (Fig. 1l and m, Tables S1 and S2). The peak fitting analysis revealed that the matrix contained a large amount of polysaccharide and a relatively small fraction of protein, with the latter being predominantly in β-sheet form. Moreover, part of the capsule matrix was undissolved in hot NaOH solution (1 M), which hydrolyzed the protein component, and its FTIR spectrum was similar to reported chitin (Figure S2) (Cuong et al. 2016), suggesting that chitin is an important constituent of the matrix. Interestingly, we found vaterite particles were present in the albumen gland and fresh eggs (see the following section in Fig. 2). These particles partially transformed into calcite even under a mild purification process simply using ddH_2_O (Fig. 1j), whereas vaterite in the egg capsule remained stable under harsh chemical treatment (NaClO), suggesting the presence of internal stabilizers.

Then we considered where the calcium carbonate in the egg capsule is sourced. Because the snail eggs cannot obtain calcium from the environment, we supposed that the female supplies calcium together with the requested nutrients. Consistent with previous studies on other *Pomacea* species, a large number of calcium carbonate particles approximately 10 μm in diameter were present in the albumen gland (Fig. 2a to c and h). According to a previous study, these particles were vaterite (Hall and Taylor 1971), which were confirmed by our XRD data (Fig. 1j). Notably, we found numerous calcium carbonate nanoparticles with a diameter of several hundred nanometers in the egg yolk of immature eggs, including those eggs in the capsule gland duct and the newly laid ones (Fig. 2d, f, and i). However, these calcium carbonate particles were absent in mature eggs 48 h after oviposition (Fig. 2g and j). EDS line mapping confirmed heterogeneous calcium distribution in mature eggs (Fig. 2k), with lower calcium levels in the yolk compared to the capsule. We propose that during oviposition, large vaterite particles in the albumen gland are dispersed into nanoparticles and transported into the egg yolk. Following oviposition, these nanoparticles are transported to the egg surface, where they assemble into the calcified capsule.

### Effect of organic matrix on calcium carbonate crystallization

**Figure 3:**
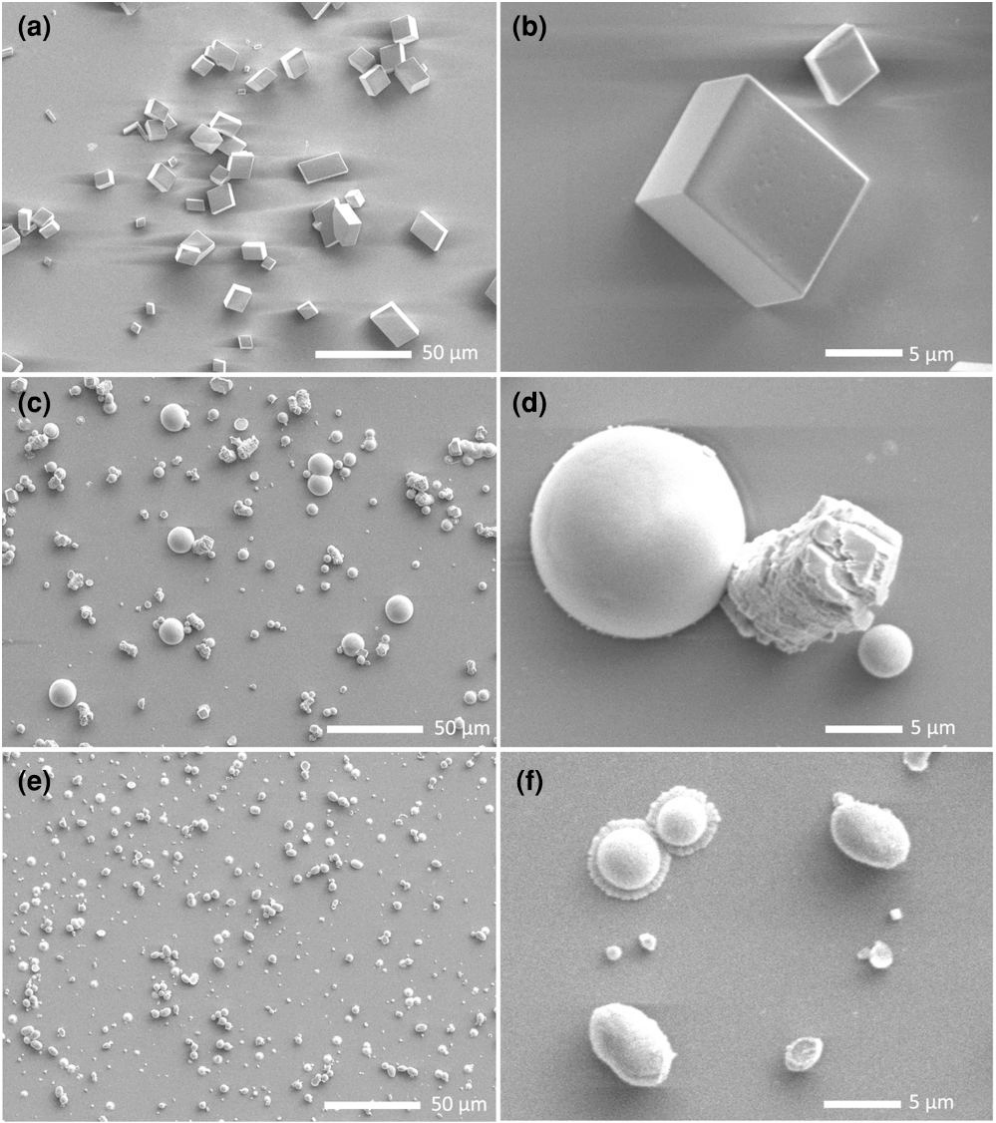
Impact of organic matrix of egg capsule on calcium carbonate crystallization. a) Control group using ddH_2_O. c) and e) Added with 0.01 g/L and 0.1 g/L organic matrix, respectively. The images in the right panel are the corresponding larger magnifications of the left panel.

As aforementioned, the vaterite in the egg capsule is quite stable, which is likely due to the embedded organic matrix. Indeed, an *in vitro* calcium carbonate crystallization experiment revealed that the organic matrix extracted from egg capsule can stabilize vaterite (Fig. 3, Figure S3). We performed an experiment in conditions similar to the snail-living freshwater environment, which favors calcite precipitation. In the control group, calcite cubes were formed (Fig. 3a and b). When 0.01 mg/L of organic matrix was added, spherical crystals similar to the vaterite in the albumen gland were precipitated, while the majority were calcite with modified morphology (Fig. 3c and d). When a high concentration of organic matrix (0.1 mg/L) was added, no calcite crystals were observed, and numerous spherical and ellipse vaterite were deposited. Moreover, the crystals had a smaller diameter and were in larger numbers. Therefore, the capsule matrix can promote vaterite formation and inhibit calcite growth.

### Proteomic analysis of the capsule proteins

**Figure 4:**
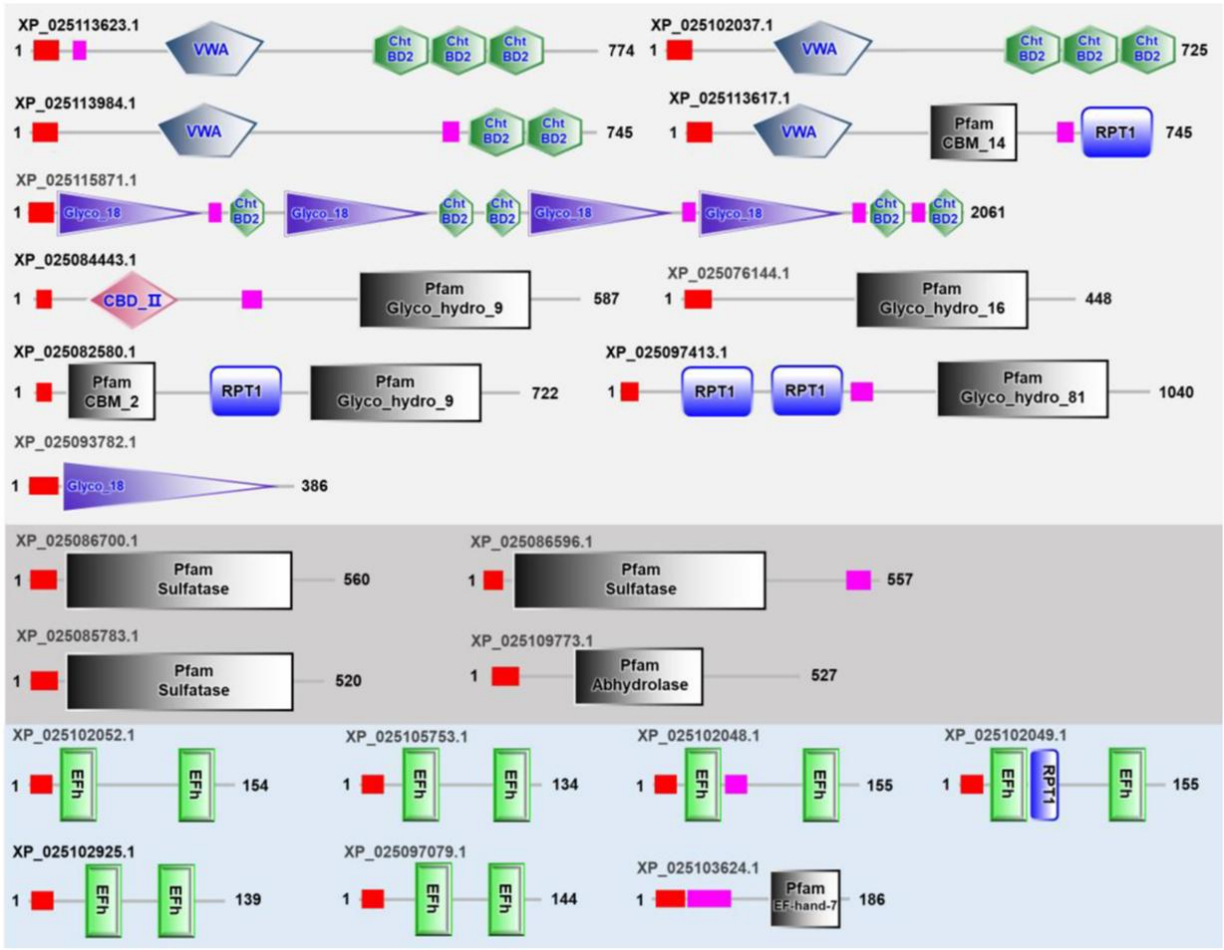
Egg capsule proteins related to biomineralization. Proteins are divided into 3 groups according to their functional domains: chitin metabolism (light gray), sulfate hydrolases (gray), and calcium-binding proteins (light blue). Red frame, signal peptide; fuchsia frame, coiled coil; VWA, von Willebrand A; ChtBD2, chitin-binding 2; RT1, tandem repeat 1; CBM, chitin-binding motif; EFh, EF hand.

As shown in the above section, the organic matrix of the egg capsule was composed of proteins and sugars. Previous studies suggest that the matrix proteins play a central role in regulating CaCO_3_ precipitation in biominerals (Huang et al. 2021; Rivera-Pérez and Hernández-Saavedra 2021). To understand how the capsule matrix is formed, we extracted the capsule proteins (Figure S4) and analyzed the protein profile by proteomics. As a result, dozens of proteins were identified (Fig. 4, Figures S5 to S8, Files S1 and S2), among which 12 were in common with shell proteins found in our previous study (Liu et al. 2023b). Although we have treated the capsules with NaClO solution thoroughly, several cytoplasmic proteins, such as actin and tubulin, were present in the capsule protein profiles (Table S3). These cytoskeletal proteins and other proteins without signal peptides were not considered for further analysis, because they are unlikely to directly participate in biomineral crystallization according to previous studies (Marie et al. 2013; Marin 2020; Huang and Zhang 2022). Domain arrangements of capsule proteins were mainly divided into 3 categories: mineralization-related proteins, immunity-related proteins, and proteins with unknown functional domains.

In the first group, half of the members were involved in chitin metabolism, such as chitin-binding and chitin degradation (Fig. 4). Particularly, 4 proteins have 1 vWA domain and 1 to 3 chitin-binding domains (defined as CBP), which is very similar to the important shell matrix protein Pif in bivalves (Suzuki et al. 2009). The concatenated vWA and chitin-binding domains indicate that CBPs may also function as a crosslinker similar to the Pif protein, interacting with other CBPs on the one hand, while linking the chitin fibers in the capsule matrix on the other hand. In addition, 4 enzymes that hydrolyze steroid sulfates and 7 calcium-binding proteins were characterized, which may be involved in organic matrix modification and calcium enrichment, respectively. Members in this group were regarded as capsule matrix proteins. The second group contained immune proteins such as immunoglobulin, lectin, protease inhibitor, and enzymes that participated in superoxide anion metabolism (Figure S6). Although these immune proteins are not directly related to CaCO_3_ deposition, they can inhibit the growth of microbes and parasites that may degrade the calcified capsules. In the third group, no conserved function domains were present in these proteins (Figure S7), so their exact roles in capsule formation remained unclear. This group, together with the immune proteins, would be regarded as capsule proteins. In addition, some proteins identified from egg capsules have conserved functional domains that were unlikely to participate in biomineralization or immunity (Figure S8). For clarity, they were excluded as capsule proteins or matrix proteins.

### Characterization of CBPs in molluscs

**Figure 5:**
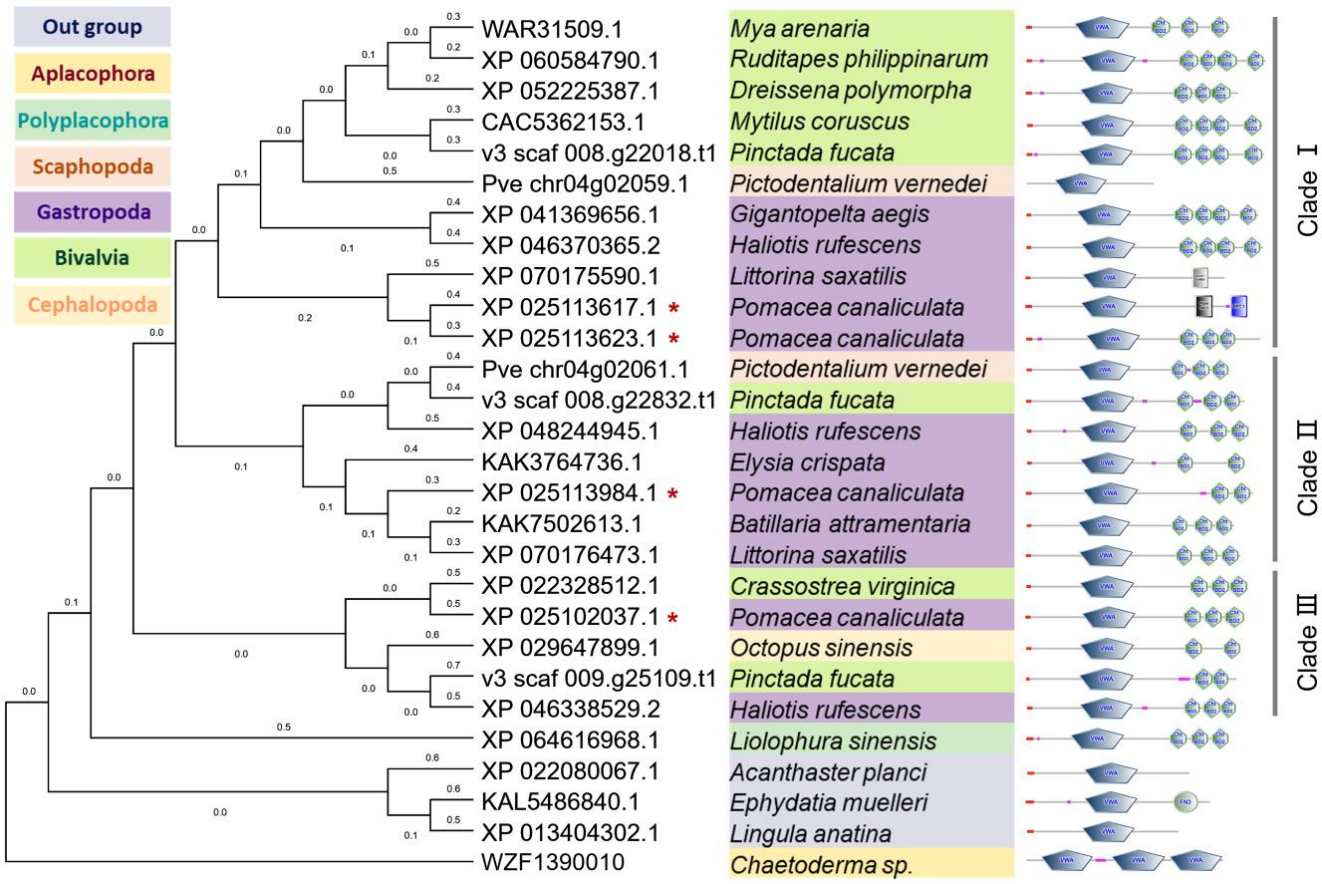
Maximum-likelihood phylogenetic tree of chitin-binding proteins (CBP) in 19 marine invertebrates, with numbers on nodes showing bootstrap values (>50%). Different classes were marked in different colors. The domain arrangement of each protein is shown on the right. The 4 *P. canaliculata* CBPs are marked with red asterisks. Since no CBP with a similar domain arrangement was found in *Chaetoderma* sp., we selected the protein with the highest homology to *Pomacea* CBPs for analysis. *Lingula anatina*, *Acanthaster planci*, *Ephydatia muelleri* were selected as outgroup species.

Since the 4 CBPs identified capsule with vWA and ChtBD domains were quite similar to the Pif (Pif97) protein, which plays a crucial role in nacre deposition, we wondered if they were orthologs. The alignment of the amino acid sequences showed relatively low identity of CBPs and Pif97 (Figure S9), while the 4 CBPs were highly homologous with each other. Then, we searched homologs of CBPs in other molluscs. Interestingly, we found homologs in all examined classes except Aplacophora, which lacks a shell structure (Fig. 5). Phylogenetic analysis grouped these CBPs into 3 clades. In clades I and II, CBPs from Scaphopoda *P. vernedei* were more closely related to bivalve CBPs, consistent with the recent genomic evidence indicating the sister taxon of Scaphopoda and Bivalvia (Song et al. 2023). Clade III was closer to the root of the tree and included a CBP from Cephalopoda. We also identified a CBP in chiton *L. sinensis* that branched from the 3 clades, following the phylogenetic tree of the mollusc phylum. Moreover, the domain arrangements of most CBPs were very similar, having a vWA in the N-terminal and 2-4 ChtBD in the C-terminal. As a comparison, homologs in the outgroup species (brachiopod *Lingula anatina*, echinoderm *Acanthaster planci*, sponge *Ephydatia muelleri*) did not have any ChtBD. This suggested that CBPs were highly conserved in molluscs and that the chitin-binding affinity of these CBPs evolved after the divergence of molluscs and other phyla.

Although chitin-mediated shell matrix assembly is prevalent in molluscs, gene expression data suggested that most of the CBPs were not related to shell biomineralization in the examined species (File S3). It should be noted that, among the 10 CBPs having gene expression data, 5 CBPs showed high expression in the gonad tissue (compared to the adductor muscle), consistent with the findings in *P. canaliculata*. Considering that 5 examined species do not produce eggs with calcified capsules, it is likely that the apple snail *P. canaliculata* recruits the existing genes to construct egg capsules.

### Transcriptome of the albumen gland and capsule gland

To discover the source of the capsule matrix, we performed a transcriptome assay of the potential organs. Because the zygote obtains the yolk and capsule gel from the albumen gland duct and capsule gland duct (Catalán et al. 2002; Dreon et al. 2006), we mainly focused on these 2 organs. The adductor muscle was chosen as a control. It should be noted that since the coiled part of the capsule gland is embedded in the albumen gland, which is technically difficult to separate, the albumen sample would contain part of the capsule gland cells.

**Figure 6:**
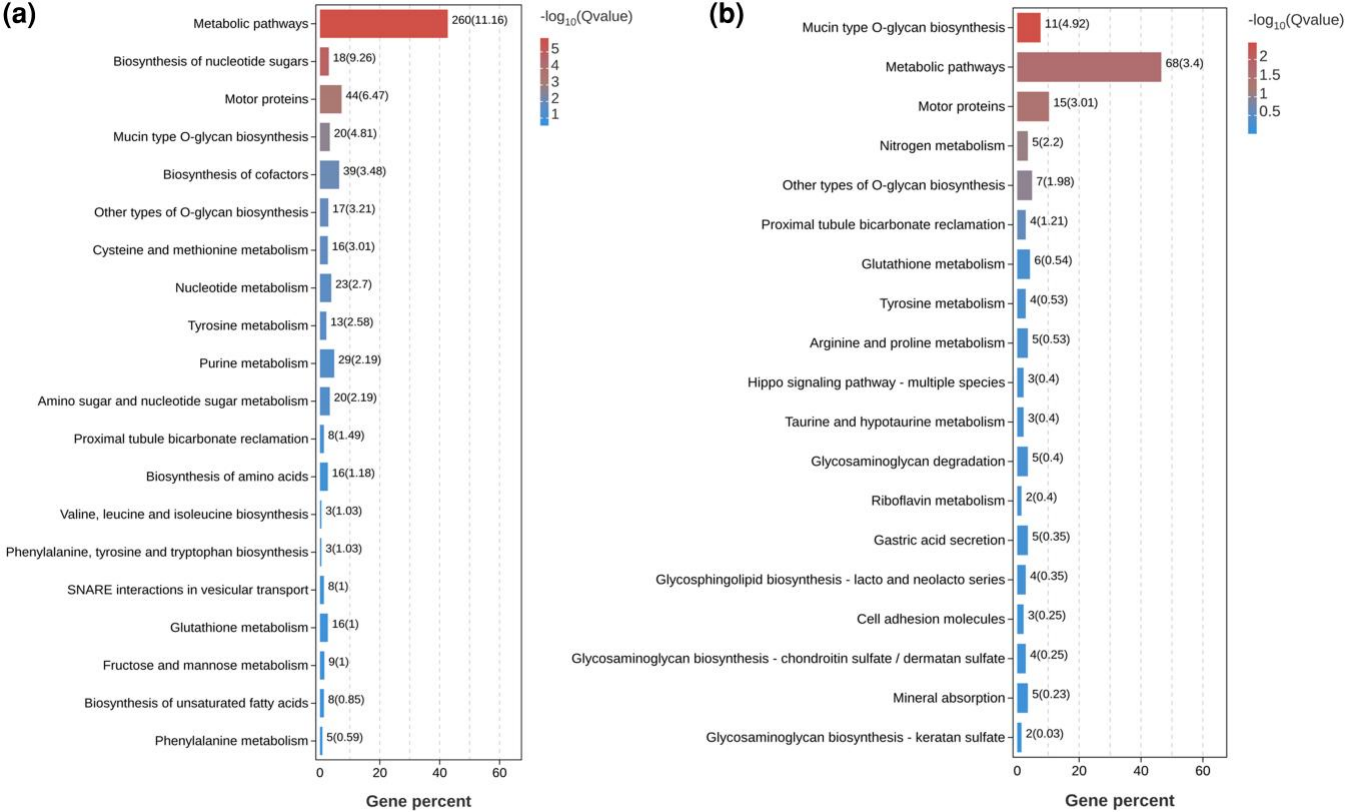
KEGG pathways of upregulated DEGs between albumen gland and adductor muscle a) and between capsule gland and adductor muscle b), respectively.

A total of 2,675 and 1,259 genes were found to be upregulated and downregulated in the albumen gland compared to the adductor muscle, respectively (Figure S10). The upregulated and downregulated genes in the capsule gland compared to the adductor muscle were 2,164 and 884, respectively (Figure S7). KEGG pathway enrichment revealed that while several pathways, including mucin type O-glycan synthesis, tyrosine metabolism, and glycosaminoglycan degradation, were enriched in both glands, some pathways were only enriched in one of the glands (Fig. 6). This is consistent with the differential secretion of the 2 glands. For example, genes involved in the biosynthesis of amino acids and nucleotide metabolism were upregulated in the albumen gland, while the capsule gland upregulated genes in mineral absorption and glycosaminoglycan biosynthesis-keratan sulfate, which directly participate in biomineralization. Furthermore, most genes coding capsule matrix proteins and capsule proteins were highly expressed in albumen gland and/or capsule gland (Figures S11 to S13), suggesting both glands were involved in capsule formation.

## Discussion

### A unique calcium transport system from the albumen gland to the egg capsule

**Figure 7.**
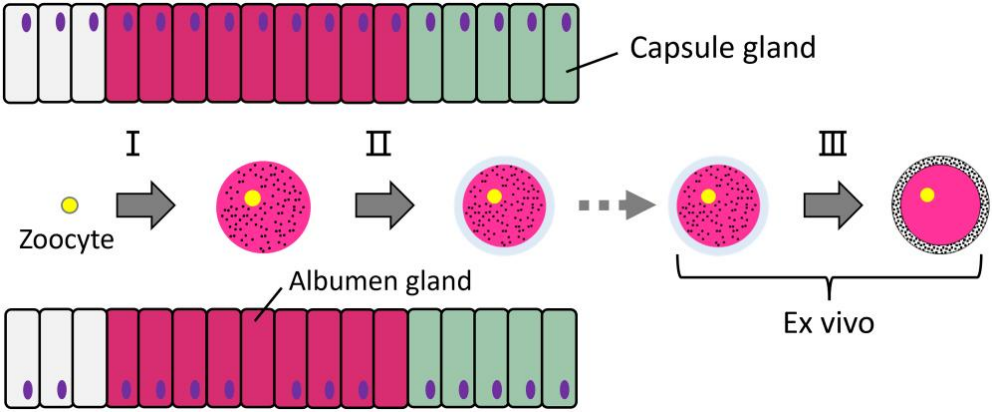
Schematic diagram of the egg capsule biomineralization. Before being laid, the zoocyte (yellow) sequentially accumulates egg yolk (red) and egg gel (gray) from the ducts of the albumen gland and the capsule gland, respectively (processes I and II). After being laid, the vaterite nanoparticles (black dots) are delivered to the egg surface and deposited as the forming capsule (process III).

The calcified egg capsule is a unique innovation of the *Pomacea* genus, contributing to the snail's highly successful reproduction and subsequent global invasion. Unlike mollusc shell formation, where calcium and carbonate ions are continuously secreted by the mantle and deposited at the mineralization interface (Tompa 1976; Lowenstam and Weiner 1989), *P. canaliculata* egg capsules become mineralized *ex vivo* after oviposition. This poses a challenge: the laid eggs cannot obtain additional calcium from the parent or the environment. Here, we found that vaterite nanoparticles were pre-stored in the yolk of pre-oviposited and freshly laid eggs (Fig. 2) and gradually depleted as capsule mineralization proceeds. This suggests a 2-phase process: (i) internal storage of vaterite nanoparticles and (ii) their subsequent transport to the egg surface for mineralization (Fig. 7). Although some terrestrial snails and marine gastropods (e.g. Neritidae) also produce calcareous egg capsules, it occurs under direct maternal regulation *in vivo* (Tompa 1976; Bigatti et al. 2010), which would face a volume limit and be time consuming. In contrast, the apple snail can lay a mass of eggs that complete calcification *ex vivo* by mobilizing the metastable vaterite stock, which is more soluble than calcite and aragonite (San et al. 2023). In this case, the female snail can safely lay a large number of unmineralized eggs in a short time; at the same time, the eggs form a protective capsule rapidly. The capsule usually becomes mineralized within 12 h, just in time to avoid desiccation and solar radiation. Therefore, calcification via vaterite transportation would endow *P. canaliculata* with a great advantage by producing large amounts of offspring terrestrially.

Then, how are these calcium carbonate nanoparticles transported? Proteomic analysis by Sun et al. (2012) identified calcium-binding proteins and actin in the perivitelline fluid, and we detected actin and tubulin in the capsule's organic matrix (Table S3), even after rigorous sodium hypochlorite treatment to eliminate cellular contaminants. We hypothesize that cytoskeletal elements facilitate nanoparticle transport, leading to co-deposition of vaterite and cytoskeletal proteins in the capsule. This is supported by the close association of calcium clusters with the cytoskeleton in albumen gland cells (Catalán et al. 2002), which secrete perivitelline fluid. However, direct evidence for cytoskeletal involvement in perivitelline fluid-mediated transport awaits further investigation.

### Stabilization of vaterite in the egg capsule

Biominerals typically contain small amounts of organic matrix, which governs the nucleation, growth, morphology, and crystal phase of the minerals (Sudo et al. 1997; Evans 2019). For instance, shell matrix proteins (Pif80, N16) from *P. fucata* stabilize aragonite and modify CaCO_3_ morphology (Ponce and Evans 2011; Bahn et al. 2017). Similarly, the organic matrix from *Pomacea* egg capsules can stabilize vaterite *in vitro*, with concentration-dependent effects on crystal morphology (Fig. 3), confirming its regulatory role in CaCO_3_ growth. The capsule's composition of pure vaterite is remarkable, as vaterite is a metastable polymorph of CaCO_3_ and is rarely found in biominerals. The selection pressure for vaterite in the apple snail capsule is unclear. This may be ascribed to the high solubility of vaterite, which allows the hatched juveniles to easily break the egg capsules. Alternatively, as aforementioned, vaterite has been selected as a rapid CaCO_3_ mobilization system in the apple snail, while the resulting vaterite capsule is a spin-off of the system.

Interestingly, we identified 4 sulfatases which hydrolyze sulfo groups from sulfated polysaccharides in the capsule matrix. Because sulfate and sulfated polymers can inhibit the vaterite-to-calcite transformation and regulate vaterite growth (Zhao et al. 2014; Balabushevich et al. 2024; Kim et al. 2025), the sulfatases may modulate vaterite deposition in the capsule by modifying the sulfated polysaccharides. Consistently, sulfated polysaccharides were found as the primary secretions of capsule gland cells (Catalán et al. 2002). In mollusc shell mineralization, sulfates were supposed to create the supersaturation necessary for nucleation and cooperate with β-sheet structured carboxylates in orienting calcite crystallization (Addadi et al. 1987). Although the FTIR spectrum did not show clear evidence of sulfated polysaccharides in the NaClO-treated capsules (Fig. 1). This absence might indicate that sulfo groups were efficiently hydrolyzed from the sugar and precipitated as CaSO_4_. Due to its crucial role in egg capsule formation, sulfatase may be a potential target for drug development to control apple snail reproduction, especially when considering that biomineralization in the native freshwater snails does not rely on sulfatases (Liu et al. 2023b).

### Evolution of a new biomineralization toolkit

The emergence of a calcified capsule in apple snails is likely coupled with the orogenesis more than 28 million years ago (Sun et al. 2019), suggesting an adaptation to the drying habitats. Compared to the exoskeleton shell that emerged in the Cambrian period more than 542 million years ago (McDougall and Degnan 2018), the capsule is a newly evolved biomineral. Notably, the protein profiles were quite different between these 2 biominerals (Figure S5). However, as indicated by the FTIR and proteomics results, the egg capsule may use chitin, a common structural component in invertebrate biominerals, to form the organic matrix cross-linked by chitin-binding proteins. In this case, the capsule matrix becomes an integrated framework and thus constructs the packed mineral layer. This chitin-mediated assembly is highly reminiscent of the organic matrix construction in molluscan shells, as shown in previous studies (Addadi et al. 2006; Marin 2020; Liu and Zhang 2021). Therefore, the biomineralization toolkit of the capsule may be derived from the conserved shell mineralization. Additionally, the abundance of calcium-binding proteins likely facilitates Ca^2+^ enrichment and calcium carbonate particle stabilization. Among them, the protein XP_025102048.1 (assigned as protein Pca_154_3.36 in the reference) has been found as a key adaptation enabling the snail's transition from underwater to terrestrial egg deposition (Sun et al. 2019).

Chitin fibers are in the center of organic matrix assembly in molluscan biomineralization. Proteins with chitin-binding domains have been found in most molluscan shells, such as Pif and BMSP (Shimizu et al. 2024). With the multiple chitin-binding domains, and usually accompanied by protein interacting vWA domains, these proteins can crosslink the chitin fibers into a network. Similarly, the CBPs identified in *P. canaliculata* egg capsules may mediate organic matrix assembly. Unlike the highly diverse Pif-like proteins, CBPs and their orthologs across molluscan lineages exhibit remarkable conservation in domain architecture (vWA + ChtBDs), implying an ancient evolutionary origin predating the divergence of major mollusc classes. Phylogenetic analysis further revealed that CBPs are widespread (except in Aplacophora), yet their functional roles seemed to be highly diverse. This evolutionary plasticity underscores how molluscs can repurpose existing proteins to innovate biomineralization strategies. While most molluscan shells rely on Pif-guided aragonite or calcite deposition (Suzuki et al. 2009; Bahn et al. 2017; Shimizu et al. 2024), *P. canaliculata* has recruited CBPs to assemble vaterite-rich capsules, a polymorph rarely employed in biominerals. The capsule's organic matrix, enriched with sulfatases and calcium-binding proteins, may further refine this process, enabling precise control over vaterite nucleation and growth (Figs 3 and 7). Such divergence suggests that novel biomineralization systems may arise from remodeling existing proteins rather than evolving entirely new ones. Intriguingly, the conserved yet flexible nature of CBPs raises the possibility that Pif-like proteins themselves may have derived from ancestral CBPs, later specializing in shell mineralization. Future studies should investigate whether CBPs and their interactors (e.g. chitin networks) function as a modular “plug-and-play” system, enabling molluscs to rapidly adapt mineralization mechanisms to ecological challenges.

## Conclusion

In this study, we revealed how vaterite formation is regulated by organic matrices and calcium transport pathways. The findings highlight the functional specialization of reproductive glands and the role of chitin-binding proteins, sulfatases, and calcium-binding proteins in stabilizing vaterite, offering potential targets for disrupting the snail's invasive reproductive success. However, the precise mechanisms of calcium nanoparticle transport from the yolk to the capsule surface require further validation, particularly whether cytoskeletal elements facilitate this process *in vivo*. Moreover, while proteomics identified key matrix components, their exact interactions in controlling crystal nucleation and growth remain unclear. Further studies are warranted to identify the vaterite-stabilizing molecules and elucidate the underlying mechanism.

## Authors’ contributions

J.H. contributed to the design of the study, carrying out the lab work, providing financial support, and drafting the manuscript. L.L. and H.Z. helped to analyze the data and revised the manuscript. R.D. participated in data processing. All authors gave final approval for publication.

## Supplementary Material

msag164_Supplementary_Data

## Data Availability

The data that support the findings of this study have been deposited in the NCBI [database name] under accession number [PRJNA1305091]. These data are currently under embargo and will be made publicly available on [2027-09-01]. Access during the embargo period can be requested from the corresponding author.

## Additional information

Sequencing reads of the transcriptome project have been deposited in the NCBI Sequence Read Archive under the study accession SRP608506.
