# Supplementary material for: Egg capsule mineralization via vaterite transportation in the invasive apple snail Pomacea canaliculata
Source: Mol Biol Evol. 2026 Jul 7;43(7):msag164. doi: 10.1093/molbev/msag164 (PMC13394694; doi:10.1093/molbev/msag164)
Supplement: msag164_Supplementary_Data [file msag164_supplementary_data.zip › Supplemetary Information-clean version R2.docx]

Title: Egg capsule mineralization via vaterite transportation in the invasive apple snail *Pomacea canaliculata*

**Author names and affiliations**

Jingliang Huang^ab^*, Li Li^c^, Haohua Zhang^a^, Ruoxi Du^d^

^a^ Southern Marine Science and Engineering Guangdong Laboratory (Guangzhou), Guangzhou, 511458, China

^b^ School of Chemical Engineering and Technology, Sun Yat-sen University, Zhuhai, 519082, China

^c^ School of Life Science, Tsinghua University, Beijing, 100084, China

^d^ Ocean University of China, Sanya Oceanog Inst, Sanya, 572025, China

**Corresponding authors**

* Corresponding author.

E-mail address:

J. Huang, starfarming09@163.com


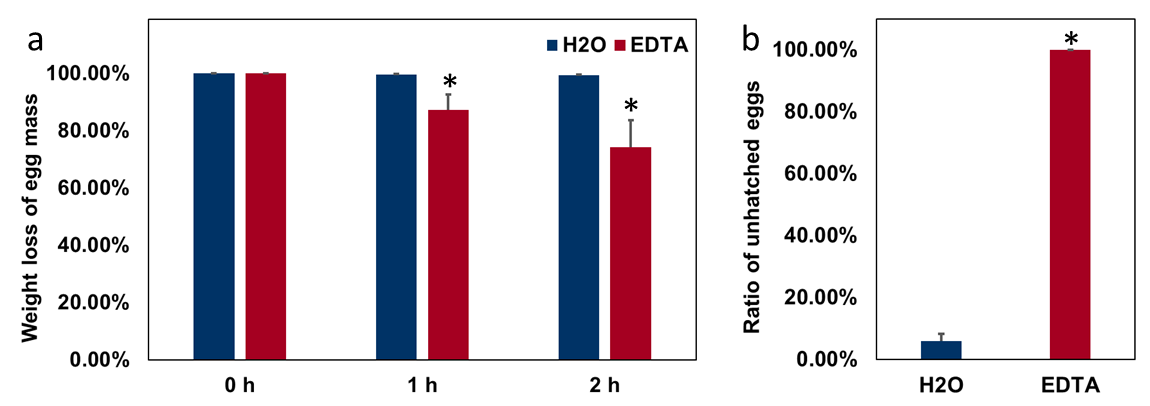


Fig. S1. Changes in weight of egg masses (a) and the proportion of unhatched eggs (b) after partial decalcification of eggshells by EDTA treatment. *, p-value < 0.01. To test the anti-evaporation effect of the egg capsule, the egg masses (<24 h post oviposition) were collected and immersed in 0.1 M EDTA for 10 min. The control group were immersed in distilled water. Then the egg masses were air dried for 12 h. For evaporation test, six samples of each group were placed in an oven and kept at 40 ℃. The weight was measured at 0, 1, and 2 h time points. For the hatching test, another six samples of each group were placed on an iron mesh. The mesh was kept in a foam box with tap water about 10 cm below the egg masses to mimic the natural hatching environment. They were then placed at room temperature for 20 days, and the hatching rate of the egg masses was calculated.


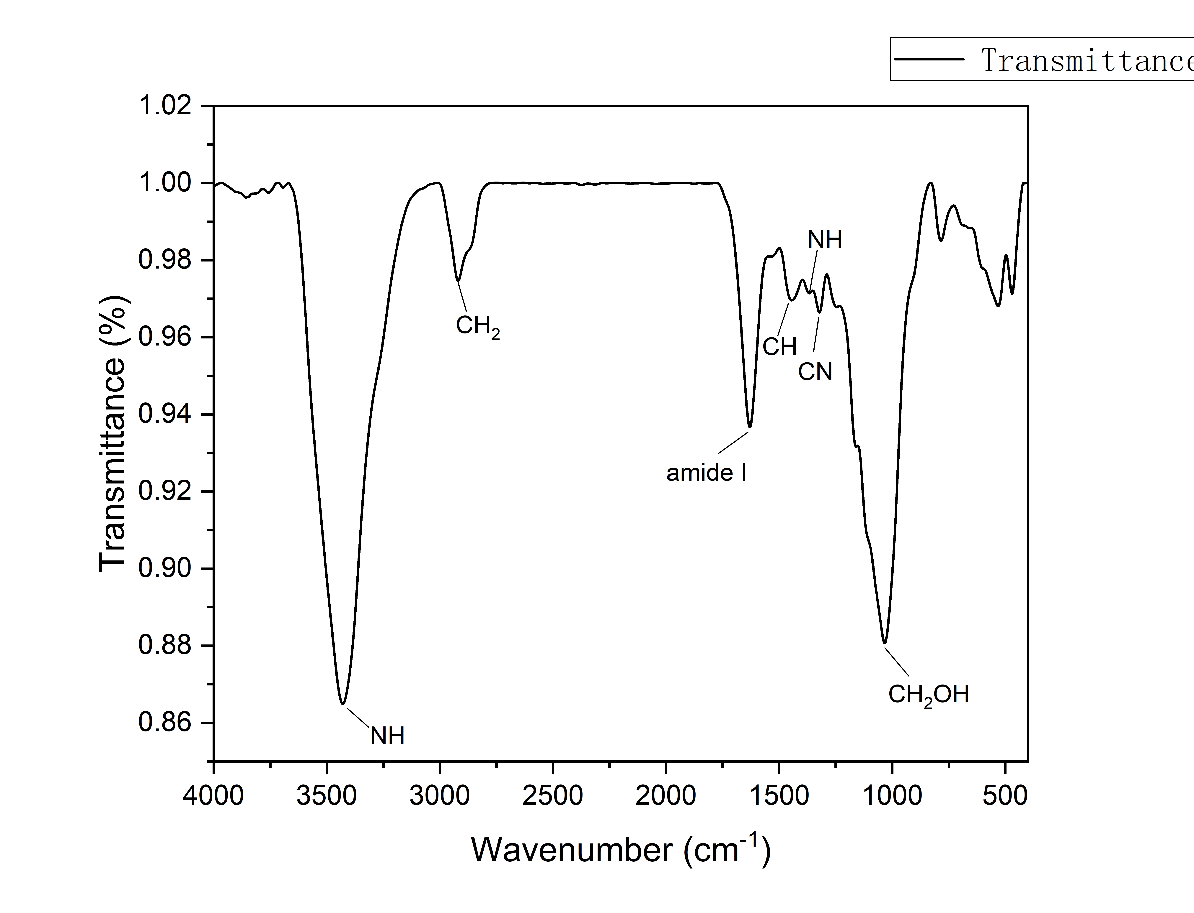


Fig. S2. FTIR spectrum of the NaOH treated EDTA-insoluble capsule matrix (EICM). EICM sample equal to Figure 1l was soaked in 1 M NaOH, and incubated at 100 ℃ for 60 min. The resultant pellets were collected by centrifugation and washed with ddH_2_O twice and then air dried. The pellets were examined by FTIR described in the Method.


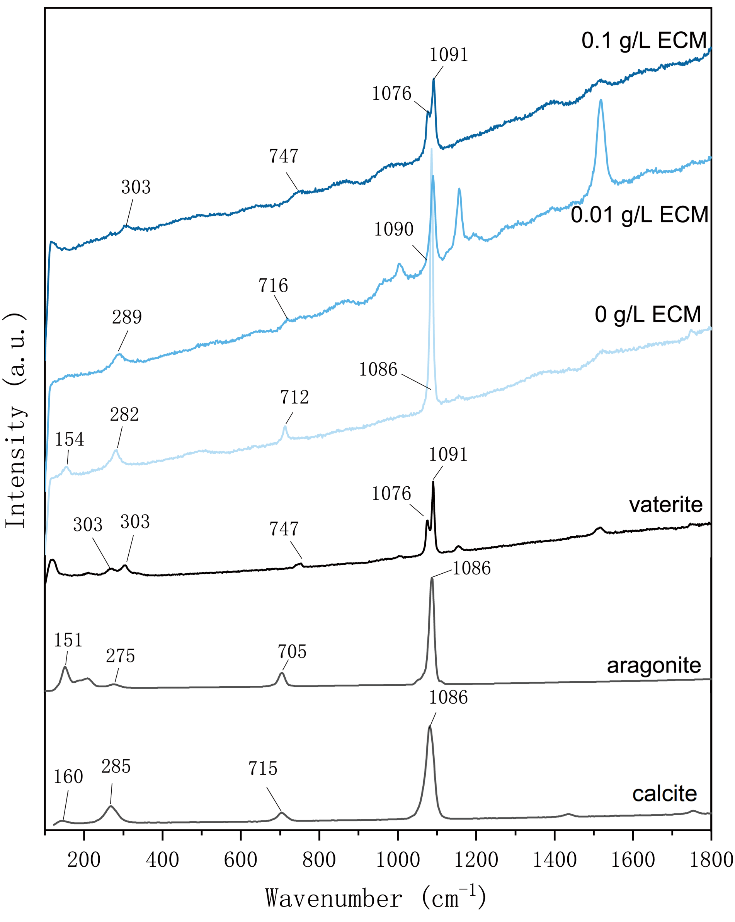


Fig. S3. Raman spectra of the crystals in the in vitro crystallization experiment, corresponding to Figure 3 in the main text. In the 0.01 g/L ECM group, the spectrum was corresponding to the spheric crystal. The calcite (RRUFFID=R050048) and aragonite (RRUFFID=R060195) standard Raman spectra were obtained from the RRUFF database. The egg capsule was used as a vaterite standard.


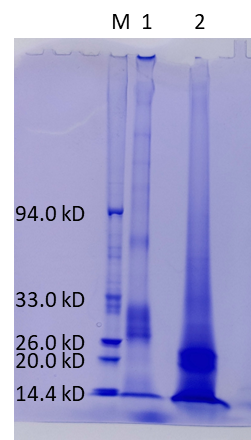


Fig. S4. SDS-PAGE electrophoresis of the extracted egg capsule proteins. M, protein ladder; 1, EDTA-soluble proteins; 2, EDTA-insoluble proteins.


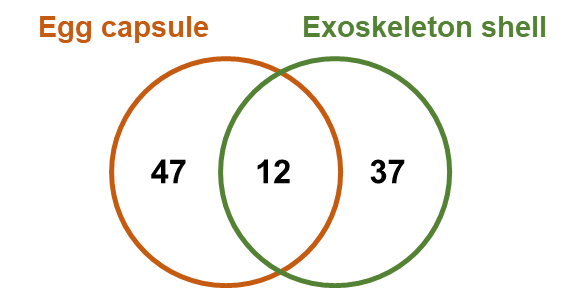


Fig. S5. Venn diagram showing the comparison of the protein profiles between the egg capsule and the exoskeleton shell.


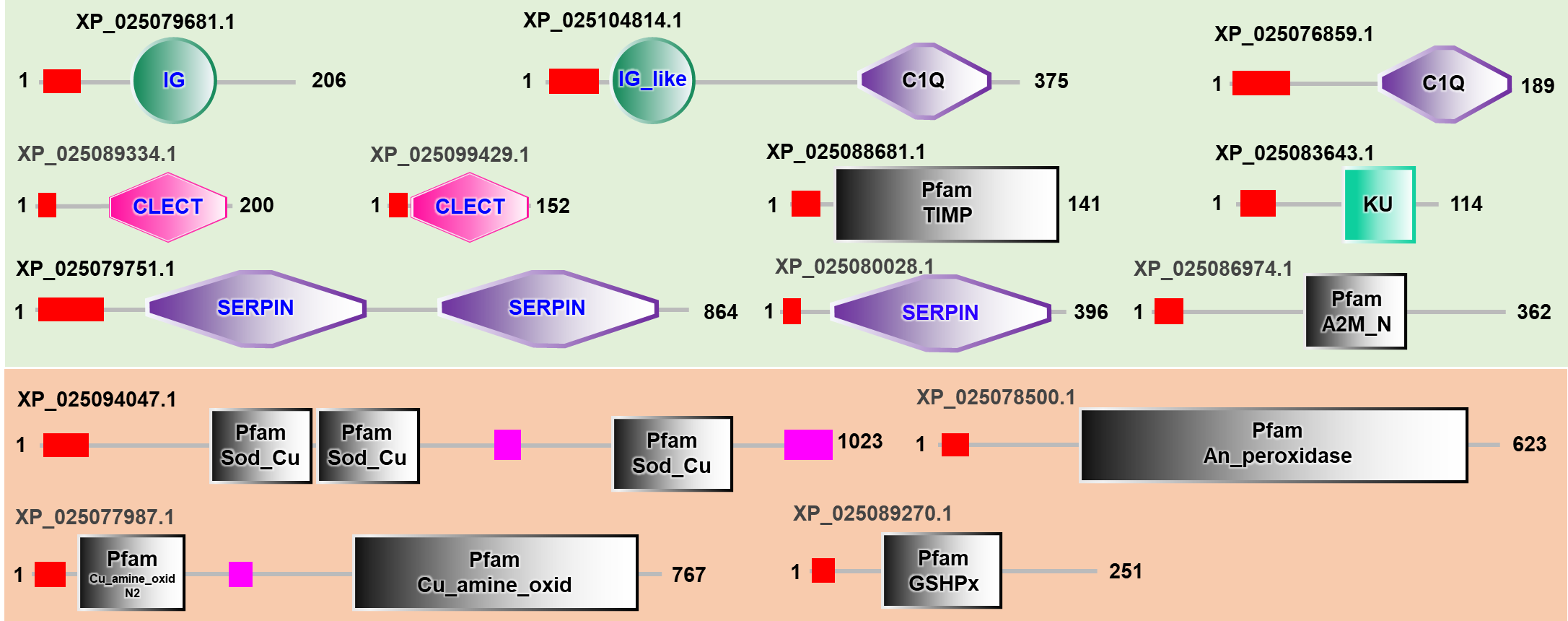


Fig. S6. Immunity-related proteins in the egg capsule. The proteins at the top row are related to cellular immunity and protease inhibitors (light green); proteins at the bottom row are redox-related enzymes, related to humoral immunity (pink). Red frame, signal peptide; fuchsia frame, coiled coil; IG, immunoglobulin G; C1Q, complement component 1q, CLECT, C-type lectin; TIMP, tissue inhibitor of metal protease; KU, kunitz-like; SERPIN, serine proteinase inhibitor; A2M, alpha-2-macroglobulin; Sod_Cu, copper superoxide dismutase; GSH-Px, glutathione peroxidase.


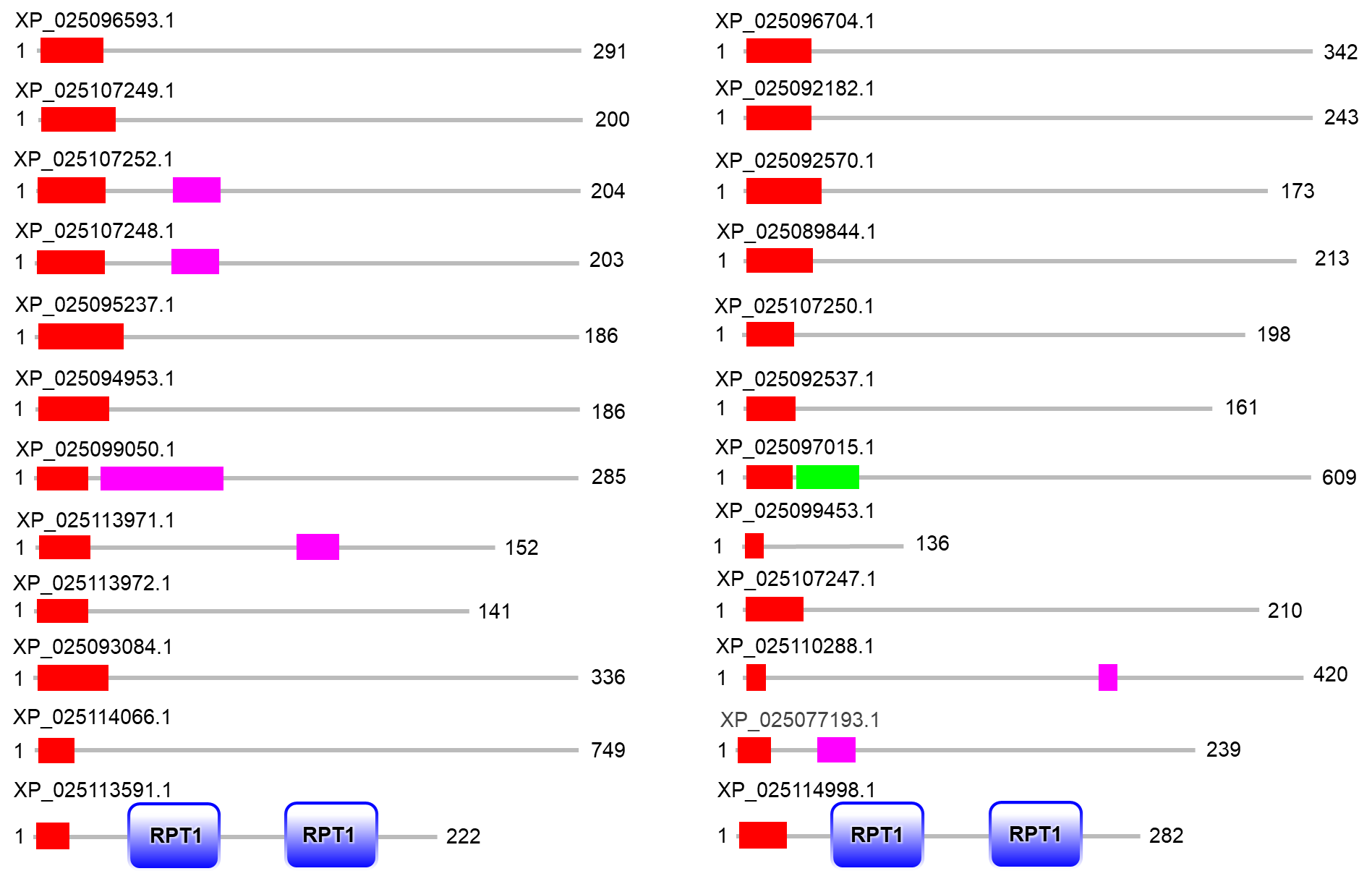


Fig. S7. Identified capsule proteins without any predicted conserved domains. Red frame, signal peptide; fuchsia frame, coiled coil; green frame, transmembrane region.


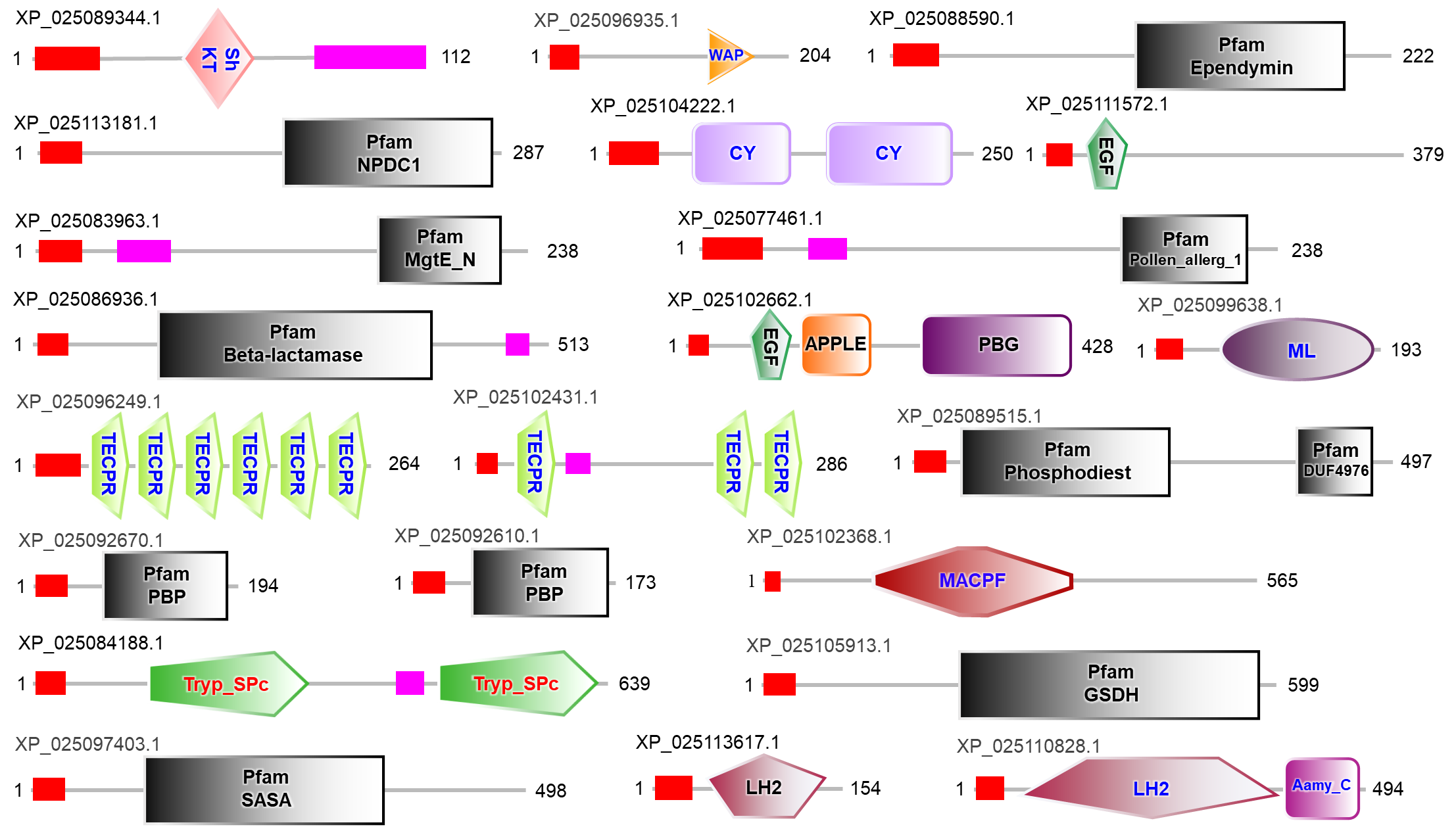


Fig. S8. Identified capsule proteins neither related to biomineralization nor to immunity. Red frame, signal peptide; fuchsia frame, coiled coil.


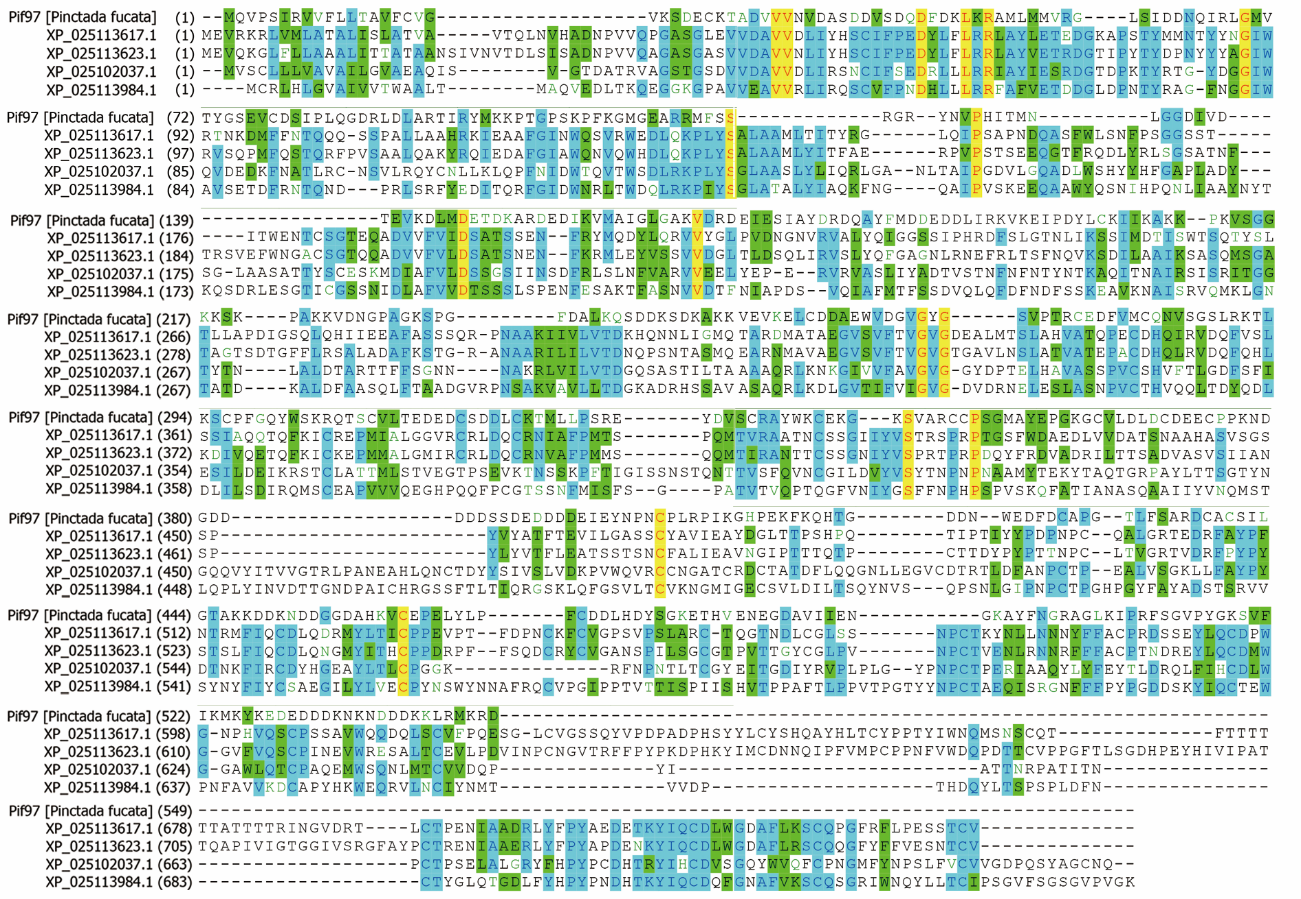


Fig. S9. Multiple alignment of the four chitin binding proteins from the capsule and the Pif97 protein from Pinctada fucata nacre.


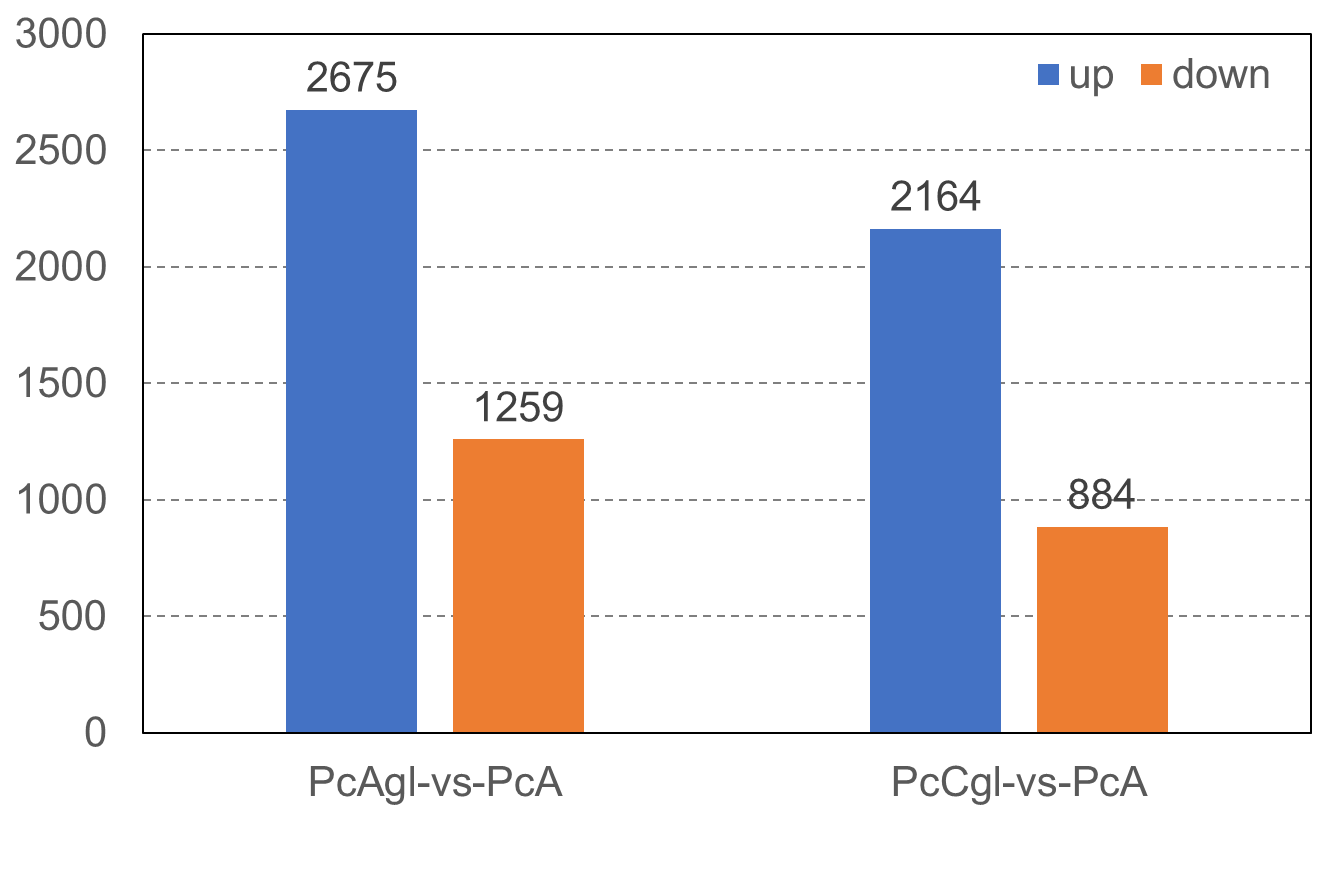


Fig. S10. Differential expressed genes in the albumen gland compared with adductor muscle (PcAgl-vs-PcA) and capsule gland compared with adductor muscle (PcCgl-vs-PcA).


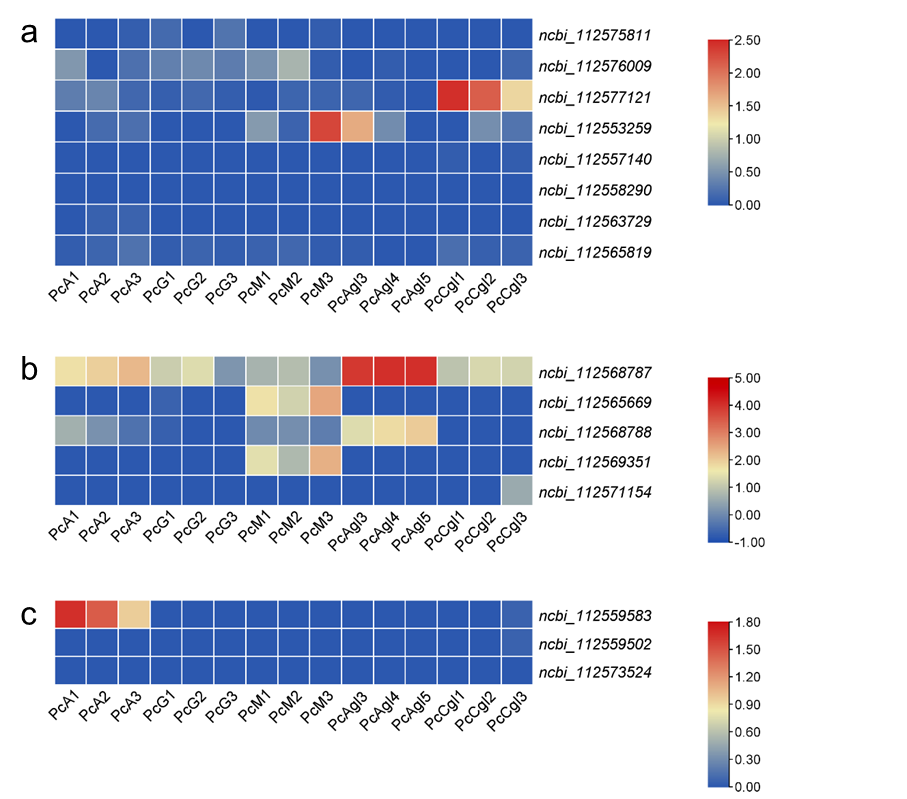


Fig. S11. Gene expression level of the biomineralization-related proteins in the egg capsule. a, chitin metabolism; b, calcium-binding; c, Sulfotase. The expression levels in b are converted into a logarithmic scale with base 10 to facilitate data presentation.


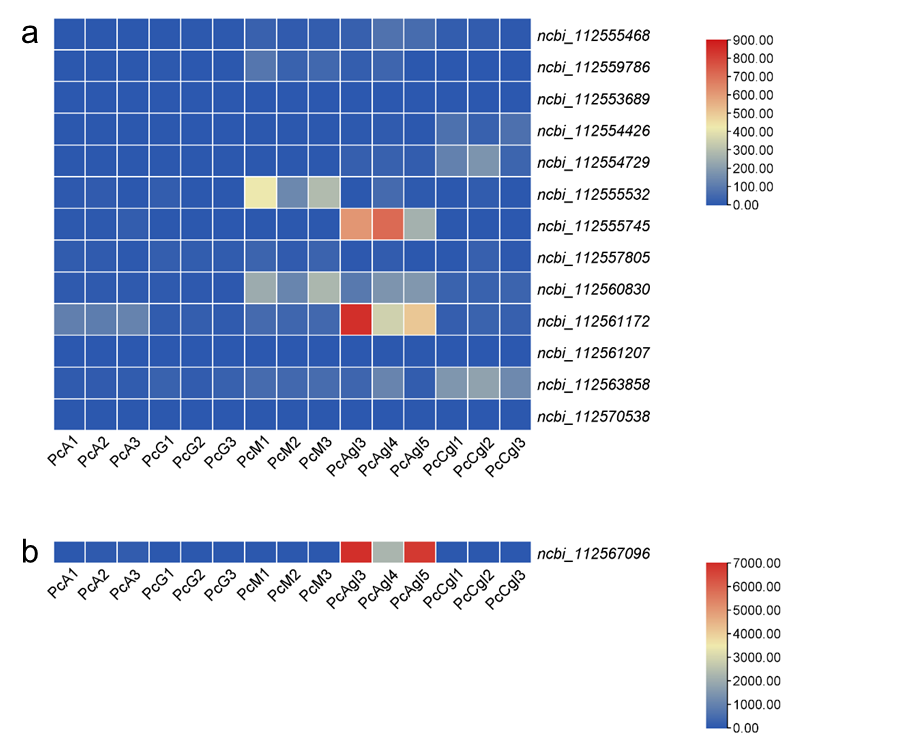


Fig. S12. Gene expression levels of the immunity-related proteins in the egg capsule. The gene ncbi_112567096 (b) were shown separately due to its extremely high expression level compared to other genes (a).


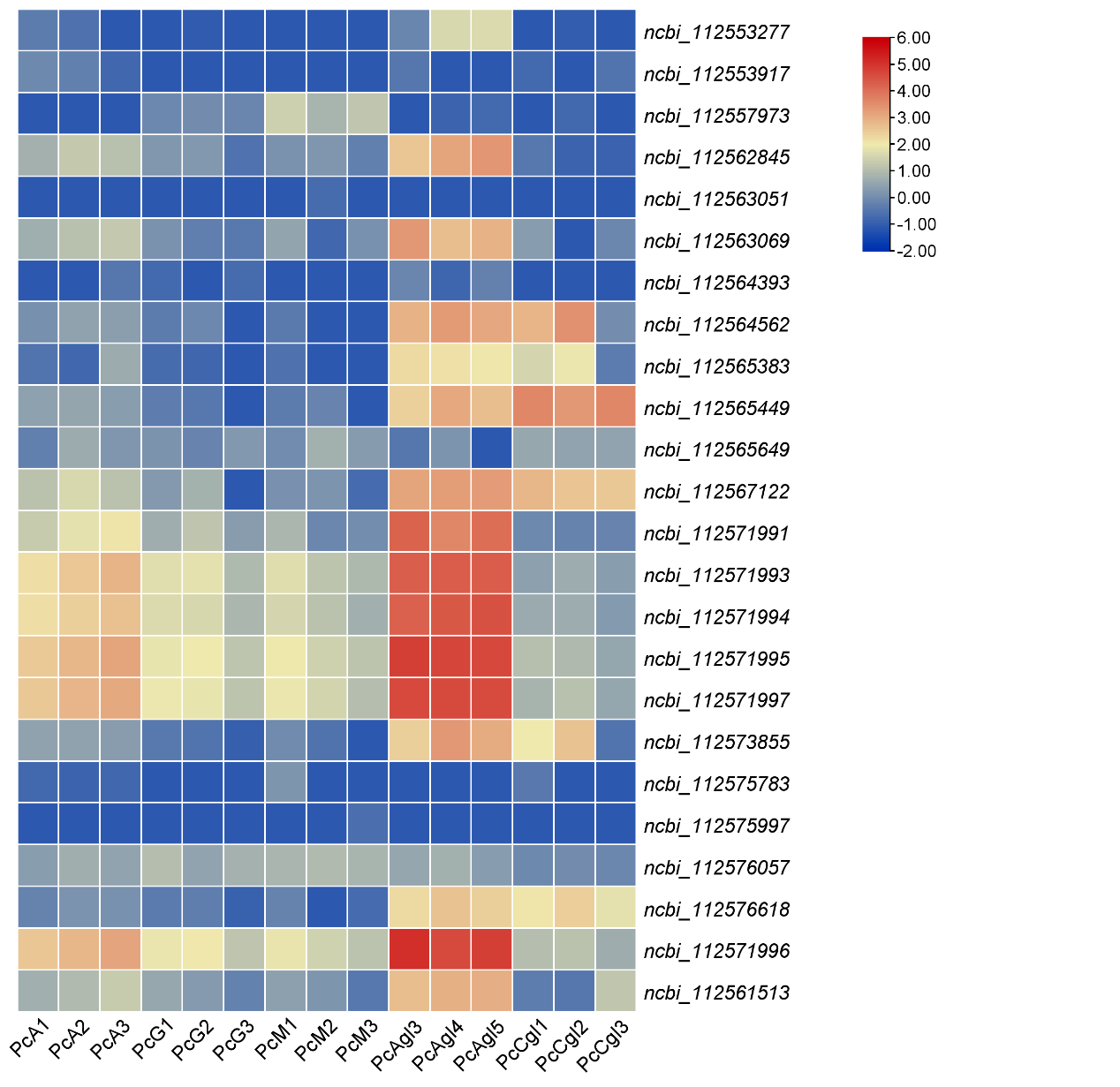


Fig. S13. Gene expression levels of those proteins without any predicted conserved domains. The expression levels of these genes are converted into a logarithmic scale with base 10 to facilitate data presentation.

Table S1. Multiple peak fit of the FTIR spectrum of the egg capsule organic matrix (region 1800-1000 cm^-1^), corresponding to Figure 1m.

|  | Parameters | Average | SD* |  | Parameters | Average | SD |
| --- | --- | --- | --- | --- | --- | --- | --- |
| 1 | Wavenumber | 1663.92 | 1.36 | 11 | Wavenumber | 1278.01 | 149.01 |
|  | FWHM | 29.83 | 4.63 |  | FWHM | 112.23 | 1088.15 |
|  | Area | 4.46 | 1.34 |  | Area | 6.41 | 213.29 |
| 2 | Wavenumber | 1635.73 | 0.81 | 12 | Wavenumber | 1234.52 | 7.40 |
|  | FWHM | 40.19 | 5.74 |  | FWHM | 58.86 | 71.30 |
|  | Area | 13.93 | 3.21 |  | Area | 6.71 | 28.01 |
| 3 | Wavenumber | 1604.82 | 1.94 | 13 | Wavenumber | 1161.09 | 3.73 |
|  | FWHM | 41.75 | 6.33 |  | FWHM | 28.92 | 20.80 |
|  | Area | 7.58 | 2.17 |  | Area | 3.33 | 6.71 |
| 4 | Wavenumber | 1552.60 | 2.00 | 14 | Wavenumber | 1140.00 | 4.51 |
|  | FWHM | 32.04 | 9.53 |  | FWHM | 31.88 | 27.60 |
|  | Area | 2.70 | 1.38 |  | Area | 4.45 | 7.29 |
| 5 | Wavenumber | 1515.02 | 3.88 | 15 | Wavenumber | 1121.27 | 4.02 |
|  | FWHM | 43.56 | 11.26 |  | FWHM | 30.93 | 22.37 |
|  | Area | 11.02 | 7.09 |  | Area | 5.06 | 10.14 |
| 6 | Wavenumber | 1489.30 | 3.30 | 16 | Wavenumber | 1081.82 | 3.57 |
|  | FWHM | 42.50 | 22.04 |  | FWHM | 31.08 | 16.76 |
|  | Area | 17.50 | 28.32 |  | Area | 9.50 | 18.27 |
| 7 | Wavenumber | 1464.67 | 9.37 | 17 | Wavenumber | 1062.66 | 8.71 |
|  | FWHM | 61.16 | 84.17 |  | FWHM | 44.29 | 80.87 |
|  | Area | 20.62 | 80.38 |  | Area | 14.11 | 67.82 |
| 8 | Wavenumber | 1423.47 | 27.96 | 18 | Wavenumber | 1040.28 | 6.88 |
|  | FWHM | 81.67 | 160.81 |  | FWHM | 45.70 | 69.50 |
|  | Area | 34.76 | 193.22 |  | Area | 18.80 | 88.84 |
| 9 | Wavenumber | 1397.12 | 6.51 | 19 | Wavenumber | 1020.79 | 15.24 |
|  | FWHM | 69.40 | 15.84 |  | FWHM | 55.32 | 18.68 |
|  | Area | 71.21 | 101.19 |  | Area | 24.07 | 49.46 |
| 10 | Wavenumber | 1317.99 | 165.27 |  |  |  |  |
|  | FWHM | 147.50 | 259.56 |  |  |  |  |
|  | Area | 26.33 | 234.04 |  |  |  |  |

*SD, standard deviation

Table S2. Assignment of the characteristic peaks in Figure 1m.

| Wavenumber | Assignment | Wavenumber | Assignment |
| --- | --- | --- | --- |
| 1663.92 | amide I β-turns of proteins | 1278.01 | collagen proteins-amide III |
| 1635.73 | β-sheet structure of amide I | 1234.52 | amide III |
| 1604.82 | polysaccharides | 1161.09 | C-O of proteins and carbohydrates |
| 1552.6 | amide II | 1140 | phosphate and oligosaccharides |
| 1515.02 | amide II | 1121.27 | carbohydrates |
| 1489.3 | deformation C-H | 1081.82 | phosphate band |
| 1464.67 | acyl chain of lipid | 1062.66 | sugar |
| 1423.47 | polysaccharides | 1040.28 | sugar moiety |
| 1397.12 | methyl groups of proteins | 1020.79 | glycogen |
| 1317.99 | collagen proteins-amide III |  |  |

Table S3. Cytoskeletal proteins in the organic matrix of the eggshell.

| Accession | Description | Score |
| --- | --- | --- |
| XP_025112424.1 | actin, adductor muscle [Pomacea canaliculata] | 43.53 |
| XP_025112942.1 | actin, cytoplasmic-like [Pomacea canaliculata] | 44.53 |
| XP_025093891.1 | LOW QUALITY PROTEIN: tubulin alpha-1A chain-like [Pomacea canaliculata] | 25.81 |
| XP_025113840.1 | tubulin alpha-3 chain-like [Pomacea canaliculata] | 18.51 |
| XP_025114330.1 | tubulin beta chain [Pomacea canaliculata] | 26.50 |
